# NF-κB-Induced Upregulation of miR-146a-5p Promoted Hippocampal Neuronal Oxidative Stress and Pyroptosis *via* TIGAR in a Model of Alzheimer’s Disease

**DOI:** 10.3389/fncel.2021.653881

**Published:** 2021-04-16

**Authors:** Bo Lei, Jiaxin Liu, Zhihui Yao, Yan Xiao, Xiaoling Zhang, Yueting Zhang, Jianguo Xu

**Affiliations:** ^1^Department of Neurosurgery, West-China Hospital, Sichuan University, Chengdu, China; ^2^Department of Neurosurgery, People’s Hospital of Leshan, Leshan, China; ^3^Medical School, Kunming University of Science and Technology, Kunming, China; ^4^Department of Burn and Plastic Surgery, 926 Hospital of People’s Liberation Army, Kaiyuan, China; ^5^Special Ward, The Second Affiliated Hospital of Kunming Medical University, Kunming, China

**Keywords:** pyroptosis, oxidative stress, NF-κB, miRNA, hippocampus, Alzheimer’s disease, TP53-induced glycolysis and apoptosis regulator

## Abstract

Alzheimer’s disease (AD) is a common neurodegenerative disorder that places a heavy burden on patients and society. Hippocampal neuronal loss is a hallmark of AD progression. Therefore, understanding the mechanism underlying hippocampal neuronal death would be of great importance for the diagnosis and treatment of AD. This study aimed to explore the molecular mechanism *via* which nuclear factor kappa β (NF-κB) promotes hippocampal neuronal oxidative stress and pyroptosis in AD. We collected serum samples from 101 healthy elderly people and 112 patients with AD at the Affiliated Hospital of Kunming University of Science and Technology between January 2017 and January 2020. Commercially available human hippocampal neurons (HHNs) were used to establish an AD model (AD-HHN) following Aβ25–35 treatment. The mRNA expression levels of NF-κB and pyroptosis markers [NLR family pyrin domain-containing 3, caspase-1, interleukin (IL)-1β, and interleukin-18] mRNA and the expression level of miR-146a-5p in the serum samples of patients with AD and AD-HHNs were determined by quantitative reverse transcription polymerase chain reaction. Oxidative stress indices (reactive oxygen species, malondialdehyde, nicotinamide adenine dinucleotide phosphate, superoxide dismutase, glutathione, and catalase) were measured by Enzyme-Linked Immunosorbent Assay (ELISA). The expression of proteins [NF-κB, TP53-induced glycolysis and apoptosis regulator (TIGAR), and pyroptosis markers] was tested by western blotting. The relationship between miR-146a-5p and TIGAR was investigated using a dual luciferase reporter gene assay. We found that NF-κB and miR-146a-5p were highly expressed, while TIGAR was low expressed in patients with AD and AD-HHNs. In addition, there was a significant positive correlation between the expression levels of NF-κB and miR-146a-5p, but a negative correlation between NF-κB mRNA and TIGAR mRNA in patients with AD, as well as miR-146a-5p and TIGAR mRNA in patients with AD. In AD-HNNs, miR-146a-5p targeted and downregulated the expression of TIGAR. Knockdown of NF-κB or overexpression of TIGAR markedly attenuated oxidative stress and pyroptosis in AD-HHNs, while concurrent overexpression of miR-146a-5p inhibited these effects. In conclusion, NF-κB-induced upregulation of miR-146a-5p promoted oxidative stress and pyroptosis in AD-HNNs by targeting TIGAR.

## Introduction

Alzheimer’s disease (AD) is a common age-related neurodegenerative disorder characterized by a functional decline (Høgh, 2017). AD has a high incidence its prevalence and mortality rates, and its treatment and care are costly, thus placing a tremendous burden on caregivers and society (Yilmaz, 2015; Alzheimer’s Association, 2016; Hodson, 2018). The hippocampus is a brain area critical for learning and memory, and it has become increasingly clear that loss of hippocampal neurons occurs in AD (Mu and Gage, 2011). The fate of hippocampal neurons is essential for the occurrence and development of AD (Richetin et al., 2015; Hu et al., 2019). Therefore, understanding the mechanism underlying hippocampal neuronal death would be of great importance for the diagnosis and treatment of AD.

AD is highly correlated with neuroinflammation and oxidative stress in the brain causing neuronal loss. Nuclear factor kappa β (NF-κB) is a proinflammatory, redox-sensitive transcription factor that plays an important role in AD (Ju Hwang et al., 2019). It also regulated hippocampal neuron apoptosis and cognitive impairment (Fang et al., 2019). Besides, NF-κB plays a key role in cytokine-induced gene expression, there was a previous study has found a consistent upregulation of several brain-enriched miRNAs (miR-9, miR-34a, miR-125b, miR-146a, and miR-155) that are under the transcriptional control of NF-kB in AD brain tissues (Zhao et al., 2014). Among the several upregulated miRNAs, miR-146a-5p (miR-146 or miR-146a) drew our attention: miR-146a-5p is deregulated in the peripheral blood of patients with AD and might serve as a diagnostic or therapeutic biomarker for AD (Gupta et al., 2017; Fransquet and Ryan, 2018). It is also involved in the progression from mild cognitive impairment to AD (Ansari et al., 2019). However, the molecular mechanism *via* which miR-146a-5p promotes progression to AD is still unknown.

Network analysis has revealed that miRNAs, including miR-146a-5p, are associated with the immune system, cell cycle, gene expression, cellular response to stress, and neuron growth factor signaling (Swarbrick et al., 2019). When we predicted the target genes of miR-146a-5p using bioinformatics, TP53-induced glycolysis and apoptosis regulator (TIGAR) drew our attention due to its role in oxidative stress in AD (Katsel et al., 2013). Therefore, we studied the role of miR-146a-5p/TIGAR in AD.

Oxidative stress contributes to AD by promoting amyloid-beta peptide (Aβ) deposition, hyperphosphorylated tau protein accumulation, and synapse and neuronal cell loss (Chen and Zhong, 2014; Tönnies and Trushina, 2017). Oxidative stress products can activate cells, induce pyroptosis, and promote disease progression (Wang et al., 2019). Pyroptosis is a recently discovered type of programmed cell death that depends on the activation of caspase-1 and the release of a large number of proinflammatory factors (Shi et al., 2017). Increasing evidence has indicated that the pyroptosis of neuronal cells is closely related to the progression of AD (Fricker et al., 2018; Han et al., 2020).

This study aimed to explore the molecular mechanism *via* which NF-κB-induced regulation of miR-146a-5p/TIGAR promotes oxidative stress and pyroptosis in AD.

## Materials and Methods

### Collection of Serum Samples

Serum samples were collected from 101 healthy elderly individuals (49 men, 52 women; mean age, 63.57 ± 5.89 years) and 112 patients with AD (59 men, 53 women; mean age, 65.13 ± 6.05 years) at the Affiliated Hospital of Kunming University of Science and Technology between January 2017 and January 2020. There was no significant difference in sex and age between the group of healthy elderly individuals and the group of patients with AD. All the participants were informed of the details of the study and gave written informed consent to participate. The study was approved by the Ethics Committee of Kunming University of Science and Technology (No. KMUST-MEC-041). All experiments were performed following the guidelines of the Helsinki Declaration.

### Cell Culture and Construction of AD Model

Human hippocampal neurons (HHNs; #P10153; Innoprot, Spain) were purchased *via* Beijing Biolead Biological Science and Technology Co., Ltd. and cultured in Dulbecco’s Modified Eagle Medium/Nutrient Mixture F-12 (DMEM-F12, #P008–2; Yaji, Shanghai, China) containing 10% fetal bovine serum (#C0257; Yaji), 2% B-27 (#PB180630; Yaji), and 0.3 mg/ml glutamine (#Q112655; Yaji) at 37°C and 5% CO_2_. After 7 days, cultured HHNs were used to establish a model of AD. Briefly, HNNs were treated with 10 μmol/L Aβ25–35 (#A4559–1MG; Sigma, USA) for 72 h, and named AD-HHNs.

### Cell Transfection

AD-HHNs were seeded at 1 × 10^5^ cells/well in a 6-well plate and maintained in a humidified incubator at 37°C and 5% CO_2_. When AD-HHNs reached 70% confluency after 24 h, cell transfection was performed. Guangzhou Ruibo Biotechnology Company, Limited (China) designed and produced si-NF-κB, miR-146a-5p inhibitor, miR-146a-5p mimics, and pcDNA-TIGAR. AD-HHN cell transfection was performed by using Lipofectamine 2000 (#11668–019; Invitrogen, Carlsbad, CA, USA) according to the manufacturer’s instructions.

### Quantitative Reverse Transcription Polymerase Chain Reaction (qRT-PCR)

The total RNA of serum samples and HHNs was isolated using the TRIzol RNA Extraction Kit (#WE0192-EYI; BioMart, Beijing, China). Isolated total RNA was reverse transcribed into cDNA using a reverse transcription kit (#QN0931-OSF; BioMart). Subsequently, SYBR Premix Ex Taq II (#RR820A; Takara, Japan) was used to perform qRT-PCR using the following steps: denaturation at 90°C for 5 min, followed by 45 cycles of denaturation at 90°C for 1 min, annealing at 60°C for 40 s, and extension at 75°C for 30 s, and a final extension at 75°C for 5 min. The relative expression of mRNA and miRNA was analyzed using the 2-ΔΔCt method. The forward and reverse primers were as follows: NF-κB F: 5′-AGCACAGATACCACCAAGACC-3′ and R 5′-GGGCACGATTGTCAAAGAT-3′; miR-146a-5p F: 5′-GGCGGTGAGAACTGAATTCC-3′ and R 5′-TTGCACTGGATACGACAACC-3′; NLR family pyrin domain containing 3 (NLRP3) F: 5′-CAGACCTCCAAGACCAGGACTG-3′ and R 5′-CATCCGCAGCCAATGAACACAC-3′; caspase-1 F: 5′-TGCCTGGTCTTGTGACTTGGAG-3′ and R 5′-ATGTCCTGGGAAGAGGTAGAAACG-3′; interleukin (IL)-1β F: 5′-ACAGATGAAGTGCTCCTTCCA-3′ and R 5′-GTCGGAGATTCGTAGCTGGAT-3′; IL-18 F: 5′-ATATCGACCGAACAGCCAAC-3′ and R 5′-TTCCATCCTTCACAGATAGGG-3′; GAPDH F: 5′-TCGACAGTCAGCCGCATCTTCTTT-3′ and R 5′-GCCCAATACGACCAAATCCGTTGA-3′; and U6 F: 5′-CTCGCTTCGGCAGCACA-3′ and R 5′-AACGCTTCACGAATTTGCGT-3′.

### Western Blotting

The total protein of HHNs was extracted using a total protein extraction kit (#BB-3101; BestBio, Shanghai, China) and the concentration of isolated total protein was measured using a BCA protein quantification kit (#BB-3401; BestBio). Next, equivalent amounts of protein were separated by 10% sodium dodecyl sulfate polyacrylamide gel electrophoresis (SDS PAGE, #YJ0014B; Yiji, Shanghai, China) and transferred onto a polyvinylidene difluoride (PVDF, #SD7966555; Yiji) membrane. Subsequently, the PVDF membrane was blocked with 5% skim milk at room temperature for 1 h, then treated with primary antibodies against NF-κB (1:1,000, #ER0815; HUABIO, Hangzhou, China), anti-NLRP3 (1:1,000, #ER1706-72; HUABIO), anti-caspase-1 (1:1,000, #ET1608-69; HUABIO), anti-IL-1β (1:1,000, #ET1701-39; HUABIO), anti-IL-18 (1:1,000, #EM170401; HUABIO), anti-TIGAR (1:1,000, #ER62495; HUABIO), and anti-GAPDH (1:1,000, #ER1901-65; HUABIO) overnight at 4°C. The next day, a secondary antibody (1:1,000, #HA1024; HUABIO) was used to treat the PVDF membrane at 37°C for 1 h. Protein bands were visualized using an ECL chemiluminescence detection kit (#BB-3501; BestBio). ImageJ was used to analyze the gray values of bands.

### Enzyme-Linked Immunosorbent Assay (ELISA)

Oxidative stress indices [reactive oxygen species (ROS), malondialdehyde (MDA), nicotinamide adenine dinucleotide phosphate (NADPH), superoxide dismutase (SOD), glutathione (GSH), and catalase (CAT)] in HNNs were measured using ELISA kits according to the manufacturer’s instructions. All ELISA kits were purchased from Wuhan Chundu Biological Science and Technology Company Limited, including human ROS ELISA kit (#CD-100411-ELISA; Chundu), human MDA ELISA kit (#CD-100334-ELISA; Chundu), human NADPH ELISA kit (#CD-101534-ELISA; Chundu), human SOD ELISA kit (#CD-100247-ELISA; Chundu), human GSH ELISA kit (#CD-101068-ELISA; Chundu), and human CAT ELISA kit (#CD-102196-ELISA; Chundu).

### Dual Luciferase Reporter Genes Gene Assay

A dual luciferase reporter gene assay kit (#11402ES60; Yeasen, Shanghai, China) was used to investigate the relationship between miR-146a-5p and TIGAR. In detail, the 3′-UTR of TIGAR wild type (TIGAR-WT) or TIGAR mutant type (TIGAR-MUT) was cloned into the pGLO luciferase vector, and transfected into HHNs, with or without miR-146a-5p mimics, using Lipofectamine 2000. After 48 h, the luciferase activity of HHNs was detected using a dual luciferase reporter assay system (Promega Corporation, Madison, WI, USA).

### Statistical Analysis

In this study, data from three independent experiments were presented as means ± standard deviation and analyzed using SPSS 19.0 software. The difference between the two groups was compared by using Student’s *t*-test, and that among groups was assessed by one-way analysis of variance (ANOVA). The correlation between NF-κB mRNA and miR-146a-5p, NF-κB mRNA and TIGAR mRNA, miR-146a-5p, and TIGAR mRNA were calculated using linear regression analysis. *P* < 0.05 was considered statistically significant.

## Results

### The Expression of NF-κB Is Correlated With miR-146a-5p in Patients With AD and AD-HNNs

**Figure 1:**
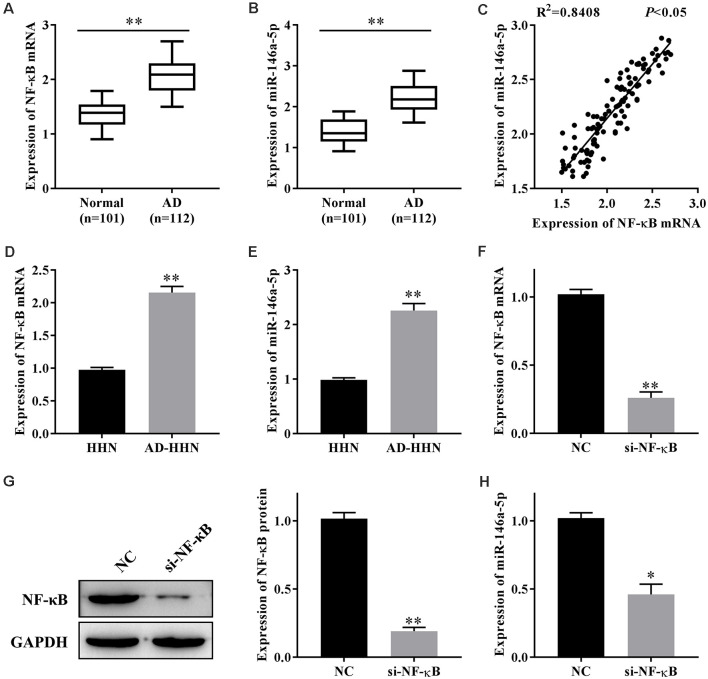
The expression of nuclear factor kappa β (NF-κB) is correlated with that of miR-146a-5p in patients with Alzheimer’s disease (AD) and a cell culture model of AD in human hippocampal neurons (AD-HNNs). **(A)** Relative expression level of NF-κB mRNA. **P < 0.01 vs. normal group. **(B)** Relative expression level of miR-146a-5p. **P < 0.01 vs. normal group. **(C)** Linear analysis between NF-κB mRNA and miR-146a-5p. **(D)** NF-κB mRNA in AD-HNNs. **P < 0.01 vs. HHN group. **(E)** NF-κB protein in AD-HNNs. **P < 0.01 vs. HHN group. **(F)** Transfection efficiency of si-NF-κB into AD-HNNs. **P < 0.01 vs. NC group. **(G)** Expression of NF-κB protein after si-NF-κB transfection into AD-HNNs. **P < 0.01 vs. NC group. **(H)** Expression of miR-146a-5p after si-NF-κB transfection into AD-HNNs. *P < 0.05 vs. NC group.

NF-κB plays an important role in AD, and miR-146a-5p is one of the target miRNAs of NF-κB. To study the function of NF-κB or miR-146a-5p in the progression of AD, we first determined their expression levels in patients with AD. qRT-PCR results showed that the expression levels of NF-κB mRNA (Figure 1A) and miR-146a-5p (Figure 1B) in serum samples from patients with AD were higher than those in healthy individuals. A linear analysis revealed a positive correlation between NF-κB mRNA and miR-146a-5p in patients with AD (Figure 1C).

Next, we established a cell culture model of AD by treating HNNs with Aβ25–35 (AD-HNNs). qRT-PCR results showed that the expression levels of NF-κB mRNA (Figure 1D) and miR-146a-5p (Figure 1E) in AD-HHNs were higher than those in HHNs. To knockdown NF-κB, we transfected si-NF-κB into AD-HHNs, and confirmed the transfection efficiency by qRT-PCR (Figure 1F) and western blotting (Figure 1G). When the transfection was successful, we determined the expression level of miR-146a-5p by qRT-PCR (Figure 1H). Knockdown of NF-κB in AD-HHNs significantly reduced the expression of miR-146a-5p. Therefore, we speculated that one way NF-κB might play a role in AD is through the upregulation of miR-146a-5p.

### NF-κB Promotes Oxidative Stress and Pyroptosis in AD-HNNs *via* miR-146a-5p

**Figure 2:**
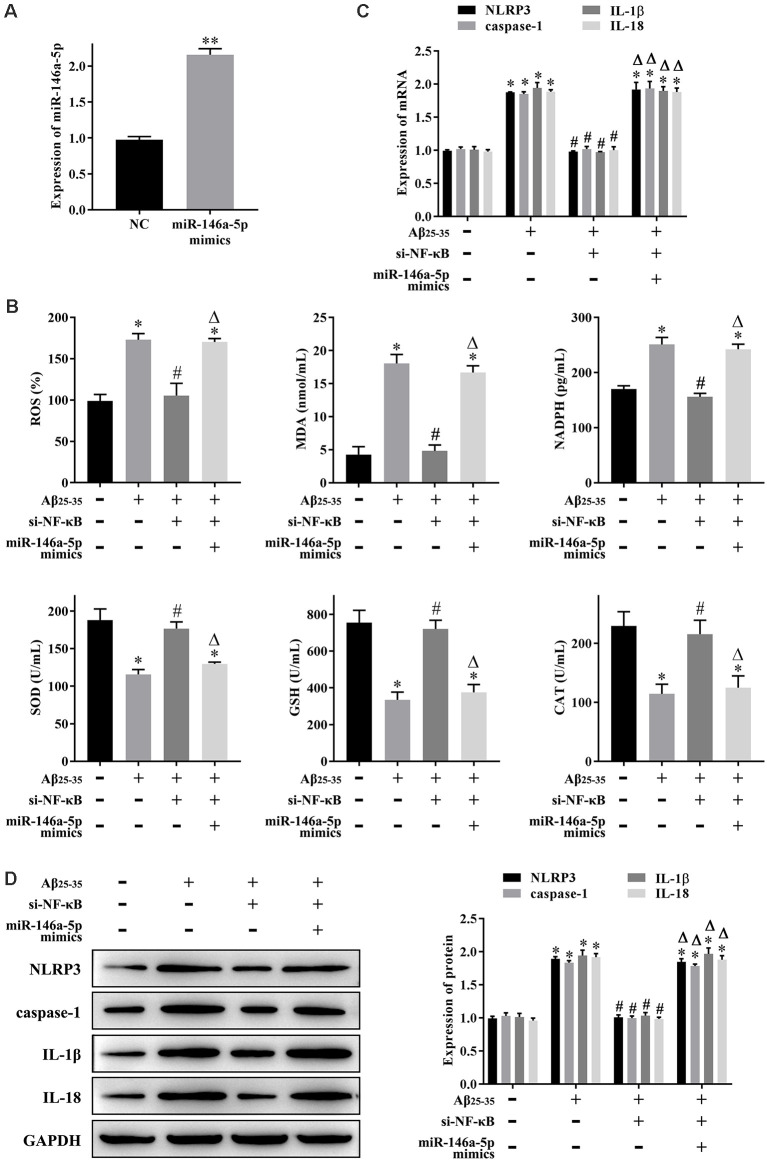
NF-κB promotes oxidative stress and pyroptosis in a cell culture model of Alzheimer’s disease in human hippocampal neurons (AD-HNNs) *via* miR-146a-5p. **(A)** Transfection efficiency of miR-146a-5p mimics into AD-HNNs. **P < 0.01 vs. NC group. **(B)** Level of oxidative stress indices [reactive oxygen species (ROS), malondialdehyde (MDA), nicotinamide adenine dinucleotide phosphate (NADPH), superoxide dismutase (SOD), glutathione (GSH), and catalase (CAT)]. **(C)** mRNA expression of pyroptosis markers (NLRP3, caspase-1, Interleukin (IL)-1β, and IL-18). **(D)** Protein expression of pyroptosis markers (NLRP3, caspase-1, IL-1β, and IL-18). *P < 0.05 vs. HHN group; ^#^*P* < 0.05 vs. AD-HHN group; ^Δ^*P* < 0.05 vs. AD-HHN+si-NF-κB group.

Hippocampal neuronal loss is a hallmark of AD progression, and oxidative stress and pyroptosis can lead to hippocampal neuronal loss. The experiments described above showed that NF-κB might play a role in AD through miR-146a-5p. Next, we studied the effect of NF-κB-induced upregulation of miR-146a-5p on oxidative stress and pyroptosis in AD-HHNs. After knockdown of NF-κB (Figures 1F,G) or overexpression of miR-146a-5p (Figure 2A) in AD-HHNs, we checked the transfection efficiency of miR-146a-5p by qRT-PCR. Next, we measured the levels of oxidative stress in each group of cells (Figure 2B). The levels of ROS, MDA, and NADPH were increased, while those of SOD, GSH, and CAT were decreased in AD-HHNs compared to HHNs. Knockdown of NF-κB attenuated oxidative stress in AD-HHNs. Moreover, knockdown of NF-κB combined with overexpression of miR-146a-5p restored all oxidative stress indices in AD-HHNs to levels similar to those in HHNs. We also measured pyroptosis markers (Figures 2C,D). The levels of NLRP3, caspase-1, IL-1β, and IL-18 mRNA and protein were increased in AD-HHNs compared to HHNs. Knockdown of NF-κB in AD-HHN reduced their expression in AD-HHNs. However, knockdown of NF-κB combined with overexpression of miR-146a-5p and elevated their expression compared to only knockdown of NF-κB in AD-HHNs. These results suggested that NF-κB might promote oxidative stress and pyroptosis in AD-HHNs through the upregulation of miR-146a-5p.

### miR-146a-5p Reduces the Expression of TIGAR

**Figure 3:**
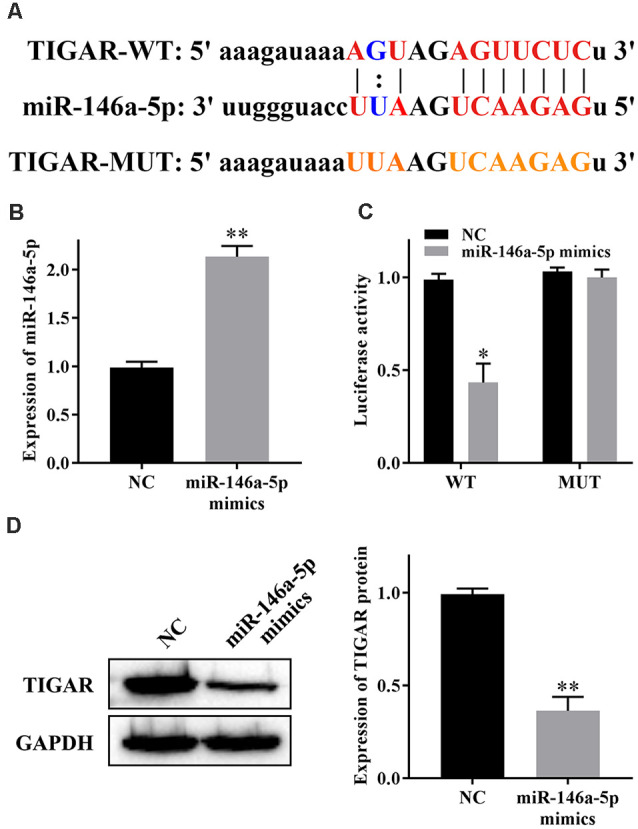
miR-146a-5p reduces the expression of TIGAR. **(A)** Binding sites between miR-146a-5p and TIGAR. **(B)** Transfection efficiency of miR-146a-5p mimics. **(C)** Dual luciferase reporter gene assay. **(D)** Expression of TIGAR protein. *P < 0.05 vs. NC group; **P < 0.01 vs. NC group.

We have previously predicted the target genes of miR-146a-5p by using bioinformatics. TIGAR aroused our great interest, and the binding sites are shown in Figure 3A. To confirm the relationship between miR-146a-5p and TIGAR, we transfected miR-146a-5p mimics into HHNs and checked the transfection efficiency by qRT-PCR. The results showed that the expression level of miR-146a-5p was substantially increased in the miR-146a-5p mimic group compared to the NC group (Figure 3B). Moreover, a dual luciferase reporter gene assay revealed that overexpression of miR-146a-5p markedly reduced the luciferase activity of TIGAR-WT but had no significant effect on TIGAR-MUT (Figure 3C). In addition, western blotting revealed that overexpression of miR-146a-5p significantly reduced the expression of TIGAR (Figure 3D). These results indicated that miR-146a-5p targeted and downregulated TIGAR in HHNs.

### miR-146a-5p Regulates Oxidative Stress and Pyroptosis in AD-HNNs *via* TIGAR

**Figure 4:**
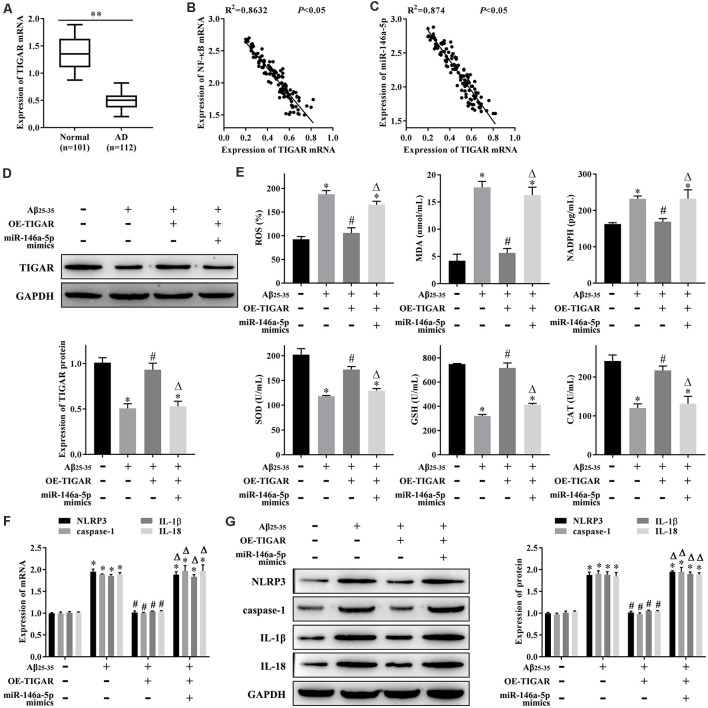
miR-146a-5p regulates oxidative stress and pyroptosis in a cell culture model of Alzheimer’s disease in human hippocampal neurons (AD-HNNs) *via* TIGAR. **(A)** Relative expression level of TIGAR mRNA. **P < 0.01 vs. normal group. **(B)** Linear analysis between NF-κB mRNA and TIGAR mRNA. **(C)** Linear analysis between miR-146a-5p and TIGAR mRNA. **(D)** Protein expression of TIGAR. **(E)** Level of oxidative stress indices (ROS, MDA, NADPH, SOD, GSH, and CAT). **(F)** mRNA expression of pyroptosis markers (NLRP3, caspase-1, IL-1β, and IL-18). **(G)** Protein expression of pyroptosis markers (NLRP3, caspase-1, IL-1β, and IL-18). *P < 0.05 vs. HHN group; ^#^*P* < 0.05 vs. AD-HHN group; ^Δ^*P* < 0.05 vs. AD-HHN+OE-TIGAR group.

To clarify whether NF-κB regulates oxidative stress and pyroptosis through the miR-146a-5p/TIGAR axis, we first measured the expression levels of TIGAR mRNA in patients with AD, and qRT-PCR results showed that it was lower in serum samples from patients with AD than that in healthy individuals (Figure 4A). The linear analysis revealed a negative correlation between NF-κB mRNA and TIGAR mRNA in patients with AD (Figure 4B), as well as miR-146a-5p and TIGAR mRNA (Figure 4C). Furthermore, we transfected pcDNA-TIGAR or miR-146a-5p mimics into AD-HHNs, then measured the expression of TIGAR protein by western blotting. The results showed that the expression of TIGAR protein in HHNs was higher than that in AD-HHNs. Transfection of pcDNA-TIGAR into AD-HHNs increased its expression. However, transfection of pcDNA-TIGAR combined with miR-146a-5p mimics into AD-HHNs resulted in an expression level of TIGAR protein similar to that of non-transfected AD-HHNs (Figure 4D). Overexpression of TIGAR relieved oxidative stress (Figure 4E) and pyroptosis (Figures 4F,G) in AD-HHNs. However, transfection of pcDNA-TIGAR combined with miR-146a-5p mimics reverted these improvements. These results indicated that miR-146a-5p exacerbated oxidative stress and pyroptosis in AD-HHNs through TIGAR.

## Discussion

AD is a neurodegenerative disorder and the most common and devastating form of dementia, which impacts the lifestyle of patients and their families, and society, due to the high costs of social and medical care (Di Resta and Ferrari, 2019). Therefore, understanding the pathogenic mechanisms of this disorder has great social, academic, and clinical implications. In this study, we explored the molecular mechanism *via* which NF-κB promotes oxidative stress and pyroptosis in AD. We found that NF-κB-induced upregulation of miR-146a-5p promoted oxidative stress and pyroptosis in a hippocampal neuronal cell model of AD through TIGAR.

NF-κB contributes to the pathogenesis of AD by participating in synaptic plasticity, learning and memory, insulin resistance, oxidative stress, neuroinflammation, and metabolism (Marwarha and Ghribi, 2017). Another study confirmed that inhibitors of NF-κB might be a novel therapeutic opportunity for AD (Seo et al., 2018). In our study, we found that NF-κB was highly expressed in patients with AD and AD-HHNs, and that knockdown of NF-κB effectively relieved oxidative stress and pyroptosis in AD-HHNs. The most well-known factor leading to AD is changes in amyloid precursor protein cleavage and production of Aβ (Soria Lopez et al., 2019). One study has proved that Aβ_25–35_ induces neuronal inflammation by reducing the nuclear import of NF-κB (Liu et al., 2017). Our results add evidence to the links between the role of NF-κB in hippocampal neurons and the progression of AD.

At the molecular level, NF-κB plays a role in AD by regulating various downstream effectors, including the molecules involved in signaling pathways, such as the PI3K/AKT, MAPK, and AGE/RAGE/GSK-3, and newly discovered noncoding RNAs (ncRNAs), which mediate neuronal toxicity or protection (Shi et al., 2016). miRNA is a type of ncRNA. In our study, we found a significant positive relationship between the level of NF-κB and miR-146a-5p in patients with AD, and knockdown of NF-κB in AD-HHNs markedly reduced the expression of miR-146a-5p. A previous study indicated that NF-κB-sensitive miR-146a-mediated modulation of complement factor H (CFH), an important repressor of the inflammatory response in the brain, regulates the inflammatory response in AD brains and human neuronal cell models of AD (Lukiw et al., 2008; Pogue et al., 2009). According to that study, NF-κB-induced upregulation of miR-146a-5p promotes the inflammatory response in AD and neuronal cells, and there exists a cause–effect relationship between inflammatory response and pyroptosis. That previous study supported our findings. It has been proven that the inflammasome platform leads to activation of caspase-1 through proximity-induced self-cleavage, which further induces the activation and secretion of IL-1β and IL-18. Activated caspase-1 also cleaves gasdermin D, which results in a particular form of lytic, programmed cell death named pyroptosis (Jorgensen and Miao, 2015; Malik and Kanneganti, 2017).

In our study, we found that overexpression of TIGAR, a target of miR-146a-5p, inhibited oxidative stress and pyroptosis in AD-HHNs. TIGAR is a p53-inducible gene that correlates with an ability to protect cells from ROS-associated apoptosis, in detail, TIGAR expression lowered fructose-2,6-bisphosphate levels in cells, resulting in an inhibition of glycolysis and an overall decrease in intracellular ROS levels, and consequently, knockdown of endogenous TIGAR expression sensitized cells to p53-induced death (Bensaad et al., 2006). A previous study has shown that TIGAR inhibits microglial pyroptosis and plays a protective role in neonatal hypoxic-ischemic brain damage; in that study, knockdown of TIGAR in rats markedly worsened pyroptosis and brain damage after hypoxia/ischemia *in vivo* and *in vitro* (Tan et al., 2021). Moreover, a study has suggested that overexpression of TIGAR increases the levels of NADPH, reduced GSH, and inducible nitric oxide synthase (iNOS), reduces intracellular ROS and increases the release of proinflammatory cytokines IL-1β and tumor necrosis factor-α in cultured primary astrocytes (Chen et al., 2018). Our work further strengthens the evidence that TIGAR affects pyroptosis through a regulated inflammatory response.

In summary, our results showed that NF-κB and miR-146a-5p were highly expressed in patients with AD and AD-HHNs and that there was a positive relationship between NF-κB and miR-146a-5p. Therefore, we speculated that one way NF-κB might play a role in AD through the upregulation of miR-146a-5p. Moreover, knockdown of NF-κB relieved oxidative stress and pyroptosis in AD-HHNs, while miR-146a-5p overexpression inhibited these effects. In addition, miR-146a-5p targeted and downregulated the expression of TIGAR in HHNs. Furthermore, the inhibition of oxidative stress and pyroptosis in AD-HHNs caused by overexpression of TIGAR was reversed by overexpression of miR-146a-5p. Therefore, our results indicated that NF-κB promoted oxidative stress and pyroptosis in AD-HHNs through the miR-146a-5p/TIGAR axis. This molecular mechanism of oxidative stress and pyroptosis in AD hippocampal neurons probably has some guiding significance for exploring new therapeutic targets, such as the knockdown of NF-κB or miR-146a-5p, overexpression of TIGAR, inhibition of oxidative stress, and pyroptosis in AD hippocampal neurons.

The major limitation of this study is that all the *in vitro* experiments were performed in normal HHNs and AD-HHNs. However, the hippocampus is more complex. It is possible that some special conditions in the hippocampus of AD patients could influence the results. Hence, we are performing further studies on this mechanism in mice with AD. In addition, it is worth noting that NF-κB plays a dualistic role in the pathogenesis of AD. In our study, we focused on the function and mechanism of NF-κB in oxidative stress and pyroptosis in a hippocampal neuronal cell model of AD. On the other hand, NF-κB might be beneficial in AD. Thus, to provide the potential pathogenesis and treatment targets of AD, more research on NF-κB in AD is warranted.

## Conclusion

This study demonstrated that NF-κB-induced upregulation of miR-146a-5p promoted oxidative stress and pyroptosis *via* TIGAR in a hippocampal neuronal cell model of AD.

## Data Availability Statement

The raw data supporting the conclusions of this article will be made available by the authors, without undue reservation.

## Ethics Statement

The studies involving human participants were reviewed and approved by Ethics Committee of Kunming University of Science and Technology. The patients/participants provided their written informed consent to participate in this study. Written informed consent was obtained from the individual(s) for the publication of any potentially identifiable images or data included in this article.

## Author Contributions

BL, JL, YZ, and JX designed the experiments. JL and ZY wrote the article. YX and XZ performed experiments and analyzed data. All authors contributed to the article and approved the submitted version.

## Conflict of Interest

The authors declare that the research was conducted in the absence of any commercial or financial relationships that could be construed as a potential conflict of interest.
